# Dietary Heme Induces Gut Dysbiosis, Aggravates Colitis, and Potentiates the Development of Adenomas in Mice

**DOI:** 10.3389/fmicb.2017.01809

**Published:** 2017-09-21

**Authors:** Marco Constante, Gabriela Fragoso, Annie Calvé, Macha Samba-Mondonga, Manuela M. Santos

**Affiliations:** ^1^Département de Médecine, Université de Montréal, Montréal QC, Canada; ^2^Nutrition and Microbiome Laboratory, Institut du Cancer de Montréal, Centre de Recherche du Centre Hospitalier de l’Université de Montréal, Montréal QC, Canada

**Keywords:** iron, heme, colitis, adenomas, microbiota, inflammatory bowel disease (IBD), colorectal cancer (CRC), red meat

## Abstract

Dietary heme can be used by colonic bacteria equipped with heme-uptake systems as a growth factor and thereby impact on the microbial community structure. The impact of heme on the gut microbiota composition may be particularly pertinent in chronic inflammation such as in inflammatory bowel disease (IBD), where a strong association with gut dysbiosis has been consistently reported. In this study we investigated the influence of dietary heme on the gut microbiota and inferred metagenomic composition, and on chemically induced colitis and colitis-associated adenoma development in mice. Using 16S rRNA gene sequencing, we found that mice fed a diet supplemented with heme significantly altered their microbiota composition, characterized by a decrease in α-diversity, a reduction of *Firmicutes* and an increase of *Proteobacteria*, particularly *Enterobacteriaceae*. These changes were similar to shifts seen in dextran sodium sulfate (DSS)-treated mice to induce colitis. In addition, dietary heme, but not systemically delivered heme, contributed to the exacerbation of DSS-induced colitis and facilitated adenoma formation in the azoxymethane/DSS colorectal cancer (CRC) mouse model. Using inferred metagenomics, we found that the microbiota alterations elicited by dietary heme resulted in non-beneficial functional shifts, which were also characteristic of DSS-induced colitis. Furthermore, a reduction in fecal butyrate levels was found in mice fed the heme supplemented diet compared to mice fed the control diet. Iron metabolism genes known to contribute to heme release from red blood cells, heme uptake, and heme exporter proteins, were significantly enriched, indicating a shift toward favoring the growth of bacteria able to uptake heme and protect against its toxicity. In conclusion, our data suggest that luminal heme, originating from dietary components or gastrointestinal bleeding in IBD and, to lesser extent in CRC, directly contributes to microbiota dysbiosis. Thus, luminal heme levels may further exacerbate colitis through the modulation of the gut microbiota and its metagenomic functional composition. Our data may have implications in the development of novel targets for therapeutic approaches aimed at lowering gastrointestinal heme levels through heme chelation or degradation using probiotics and nutritional interventions.

## Introduction

Availability of nutrients within the gut is a major driver of the microbial community structure (Kamada et al., 2013). One such nutrient is iron. In the gut, bacterial survival strongly depends on their ability to capture iron (Weinberg, 2009). For many bacterial pathogens, iron is often the limiting factor for colonization and infection (Becker and Skaar, 2014). Bacterial iron-management strategies, including iron uptake mechanisms, can occupy a large fraction of the bacterial genome (Andrews et al., 2003). Bacterial iron acquisition systems include direct contact between the bacterium and exogenous iron sources as well as more sophisticated systems that rely on molecules synthesized and released by bacteria into the extracellular medium to scavenge iron or heme from various sources. These include high-affinity iron-siderophore and heme acquisition systems, which enable bacteria to compete for iron (Wandersman and Delepelaire, 2004). As such, intestinal luminal iron availability is highly dependent on dietary components and/or iron supplementation and holds the potential to directly affect microbial composition.

Previously, we have shown that in mice, iron differentially altered the gut microbiota composition depending on the iron formulation (ferrous sulfate, ferrous bisglycinate, and ferric ethylenediaminetetraacetic acid) that was present in the diet (Constante et al., 2017). However, in addition to capturing non-heme iron, many bacteria are also proficient at capturing heme iron.

Bacterial heme acquisition systems are comprised of a series of proteins to sense and signal the up-regulation of the heme transport proteins, bind heme with high affinity, and translocate heme into the cytoplasm (Tong and Guo, 2009). Bacteria have diverse heme transport systems that include direct uptake of heme or heme proteins (e.g., hemopexin, hemoglobin, hemoglobin–haptoglobin), hemophore-mediated heme uptake, or bipartite heme receptors consisting of a two-protein system (Tong and Guo, 2009). Bacterial pathogens are particularly efficient at capturing heme and at thriving in heme-rich environments (Cescau et al., 2007; Reniere et al., 2007; Wilks and Burkhard, 2007; Tong and Guo, 2009).

Heme iron levels in the luminal gut is likely to profoundly impact on the microbial community structure. The impact of heme on the gut microbiota composition may be particularly relevant in chronic inflammation, such as found in patients with inflammatory bowel disease (IBD) comprising ulcerative colitis (UC) and Crohn’s disease (CD). During inflammation, the host responds by sequestering iron in a process that has been coined as “nutritional immunity” (Weinberg, 1977; Diaz-Ochoa et al., 2014). This process increases competition for iron and favors the growth of bacteria that are better equipped to acquire iron from diverse sources. In fact, gut dysbiosis has been consistently reported in IBD patients (Manichanh et al., 2006; Scanlan et al., 2006; Frank et al., 2007; Peterson et al., 2008; Walters et al., 2014; Winter and Baumler, 2014).

Inflammatory bowel disease patients are at increased risk of developing colorectal cancer (CRC) (Kim and Chang, 2014). Epidemiological studies have shown that CRC has a strong association with meat consumption in the Western diet (Armstrong and Doll, 1975; Sinha et al., 2009; De Stefani et al., 2012) that manifests in a dose-dependent manner (Sandhu et al., 2001; Norat et al., 2002; Larsson and Wolk, 2006). This association is further reinforced by the fact that the incidence of CRC is rapidly increasing in developing countries adopting Western-style diets (Kim et al., 2013). In UC patients, ingestion of heme-rich red meat increases the likelihood of flare relapse (Jowett et al., 2004).

In this study, we investigated the influence of dietary heme on the microbiota and inferred gut metagenome functional composition, as well as its impact on colitis and colitis-associated adenoma development in mice.

## Materials and Methods

### Animals

C57BL/6 mice were bred and maintained under standard 12:12 h light/dark conditions at the Centre de Recherche du CHUM (CRCHUM) and were co-caged at four to five mice per cage. All procedures were performed in accordance with the Canadian Council on Animal Care guidelines after approval by the Institutional Animal Care Committee of the CRCHUM.

### Diets

To study the impact of diets on microbiota, mice were fed *ad libitum* a control diet containing 50 mg/kg of iron in the form of iron sulfate (Teklad TD.120515) or a heme-supplemented diet with 50 mg/kg iron in the form of hemin (Teklad TD.120516) for 4 weeks. For the chronic azoxymethane (AOM)/dextran sodium sulfate (DSS) experiments for adenoma formation, mice were fed a control (TD.140855) diet or a diet supplemented with 25 mg/kg of iron in the form of heme (TD.140856). Diet compositions are detailed in **Supplementary Table S1**.

### Animal Treatments

*Dextran sodium sulfate (DSS)-induced acute colitis:* Colitis was induced by administering DSS (0.75% w/v of 40 000 molecular weight DSS; TdB Consultancy AB, Uppsala, Sweden) in drinking water to 20–25 g female mice for 10 days (Chassaing et al., 2014). Mice received *ad libitum* a control diet containing 50 mg/kg of iron in the form of iron sulfate (Teklad TD.120515; Envigo, Indianapolis, IN, United States) or hemin (Teklad TD.120516) starting 1 week before a cycle of 10 days of DSS. Alternatively, mice were fed *ad libitum* the control diet containing 50 mg/kg of iron in the form of iron sulfate (Teklad TD.120515) starting 1 week before DSS-treatment. For intraperitoneal hemin administration, we used the same method as reported by Zhang L. et al., 2014. Hemin was dissolved in 0.2 mol/l NaOH, titrated to pH 7.4 with HCl, and then diluted with phosphate-buffered saline (PBS). Mice were intraperitoneally administered vehicle or 75 μmol/kg of hemin (Sigma-Aldrich) 2 days before DSS-treatment.

*Azoxymethane (AOM)/DSS model:* Colitis-associated adenoma formation was induced by intraperitoneal injection of 10 mg/kg of AOM in 20–25 g female mice (De Robertis et al., 2011) that received *ad libitum* a control diet or a diet supplemented with 25 mg/kg of iron in the form of heme starting 2 weeks before AOM injection. Three days after AOM injection, mice were subjected to three cycles of 2% DSS for 5 days, followed by a recovery period of 14 days. After the third cycle, the drinking water was administered without DSS for four additional weeks.

### Histological Scoring

Colon paraffin sections were stained with hematoxylin and eosin, then subjected to blind analysis and scored. *Inflammation score:* presence of occasional inflammatory cells in the lamina propria (assigned a value of 0); increased numbers of inflammatory cells in the lamina propria (value of 1); confluence of inflammatory cells, extending into the submucosa (value of 2); and transmural extension of the infiltrate (value of 3) (Jia et al., 2008). *Tissue damage score:* no mucosal damage (value of 0); lymphoepithelial lesions (value of 1); surface mucosal erosion or focal ulceration (value of 2); extensive mucosal damage; and extension into deeper structure (value of 3) (Jia et al., 2008).

### Quantitative Reverse Transcriptase-Polymerase Chain Reaction (qRT-PCR)

Total RNA from homogenized colonic tissue samples was isolated using TRIZOL (Invitrogen, Burlington, ON, Canada). To avoid inhibition by DSS of downstream reactions, we further purified the mRNA using a second clean-up with the Qiagen mRNA Isolation Kit (Qiagen, Mississauga, ON, Canada). Using this clean up we do not find differences in β*-actin* mRNA levels between the DSS-containing samples and non-DSS-containing controls. Reverse transcription was performed with the Thermoscript RT-PCR System (Invitrogen, Burlington, ON, Canada). *Lcn2*, β*-actin*, and *interleukin-6* (*Il-6*) mRNA levels were measured by real-time PCR in a Rotor Gene 3000 Real Time DNA Detection System (Montreal Biotech, Kirkland, QC, Canada) with QuantiTect SYBRGreen I PCR Kits (Qiagen, Mississauga, ON, Canada) as described (Huang et al., 2009; Layoun and Santos, 2012). The following primers used were: β*-actin*, 5′-TGTTACCAACTGGGACGACA-3′ and 5′-GGTGTTGAAGGTCTCAAA-3; *Lcn2*, 5′-CCCATCTCTGCTCACTGTCC-3′ and 5′-TTTTTCTGGACCGCATTG-3′; *Il-6*, 5′-TGTGCAATGGCAATTCTGAT-3′ and 5′-CCAGAGGAAATTTTCAATAGGC-3′. All primers were designed to include at least one intron to allow DNA contamination detection by melting curve analysis. Relative quantitation was performed using standard curves constructed from serial dilutions of PCR products as previously described (Makui et al., 2005). Expression levels were normalized to the housekeeping gene β*-actin*.

### Heme Quantification

Heme from feces was assayed by fluorescence according to van den Berg et al. (1988). Briefly, colonic contents from mice were immediately snap-frozen and kept at -80°C until dilution in water 1:1 (w/w). Samples were homogenized, then centrifuged at 1 500 × *g* for 10 min. To 10 μl of the supernatant 200 μl of glacial acetic acid was added and mixed. Subsequently, 10 μl of freshly prepared aqueous solution of FeSO_4_.7H_2_0 (0.12 mol/l) and HC1 (4.5 mol/l) was added. Samples were immediately incubated at 60°C for 30 min after which 50 μl of the sample was added to 100 μl of 1:1 2-propanol/water (v/v). Fluorescence was measured at excitation 400 nm and emission 594 nm.

### Fecal Butyrate Quantification

Butyrate was measured at the CRCHUM Metabolomics core facility by liquid chromatography-mass spectrometry using a protocol adapted from Han et al. (2015). Briefly, samples were homogenized in 50% aqueous acetonitrile containing 2,2-dimethylbutyric acid as internal standard. After centrifugation, water-soluble carbonyl groups found in supernatants were derivatized using 3-nitrophenylhydrazine to produce the corresponding phenylhydrazone derivatives. Derivatized short-chain fatty acids (SCFAs) were separated by high-performance liquid chromatography (Shimadzu Nexera X2 UHPLC System, Columbia, MA, United States) using a Poroshell 120 EC-C18, 2.1 × 75 mm, 2.7 μm particles column (Agilent Technologies, Santa Clara, CA, United States) coupled to a guard column, and a gradient mobile phase composed of formic acid in water (solvent A) and formic acid in acetonitrile (solvent B). Detection was performed after electrospray ionization on a Sciex 6500 mass spectrometer operated in negative-ion mode.

### DNA Extraction and Illumina MiSeq Sequencing

Total DNA was extracted from snap-frozen colonic content kept at -80°C with the Powersoil DNA Extraction Kit (MO BIO Laboratories, Carlsbad, CA, United States). The amplicon library preparation and sequencing for the stool microbiota analysis were performed by the Genome Quebec Innovation Center. Briefly, amplicon libraries were constructed with the bacterial/archeal PCR primers 347F and 803R, which target the V3-V4 region of the 16S ribosomal RNA (rRNA) gene. A second PCR was performed to attach sample barcodes and the adaptor sequences used by the Illumina sequencing systems. Samples were normalized based on Quant-iT^TM^ PicoGreen^®^ dsDNA Assay Kit (ThermoFisher Scientific, Nepean, ON, Canada), pooled and purified with Agencourt AMPure beads (Beckman Coulter Canada Inc., Mississauga, ON, Canada). After quality check by DNA quantitation, real-time quantitative PCR, and microfluidic gel electrophoresis, the library was sequenced in the MiSeq system (Miseq v2 Reagent Kit, 500 cycles PE; Illumina, San Diego, CA, United States), spiked with 20% PhiX (Illumina, San Diego, CA, United States).

### Microbiota Analysis

*Pre-processing of sequence reads:* Forward and reverse 16S rRNA gene sequences obtained from Illumina (available at the Sequence Read Archive SUB2963298) were aligned to each other using the Paired-End Read (PEAR) merger (Zhang J. et al., 2014). *Clustering of reads into operational taxonomic units (OTUs):* Using the Quantitative Insights into Microbial Ecology (QIIME; Caporaso et al., 2010) software, the merged sequences were aligned to the Greengenes database version 13.8 (i.e., “closed reference” approach to clustering) containing the sequences for OTUs devoid of chimeric sequences. We used the 97% similarity database to identify bacteria at the species level. OTUs were filtered to remove taxa present in only one sample and taxa with less than 100 reads across all samples. Samples were confirmed to have a minimum of 1000 reads each.

*α- and β-diversity analysis:* The Shannon index of diversity was calculated using the R package vegan (Oksanen et al., 2015). To investigate the level of differences between experimental groups (β-diversity or how taxa are shared between groups), we performed Principal Coordinate Analysis (PCoA) using the unweighted UniFrac distance (Lozupone and Knight, 2005), with the R package phyloseq (McMurdie and Holmes, 2012), and analysis of variance (ANOVA) using distance matrices (Adonis) using the R package vegan (Oksanen et al., 2015).

*Functional analysis*: To infer sample metagenomes from the 16S rRNA gene analysis (i.e., infer the genes present in the microbiota population), we used the Phylogenetic Investigation of Communities by Reconstruction of Unobserved States tool (PICRUSt) (Langille et al., 2013) with the Greengenes version 13.5 precalculated files for the Kyoto Encyclopedia of Genes and Genomes (KEGG) genes and pathways (Kanehisa and Goto, 2000). Since the Greengenes OTU database does not change from version 13.5 to 13.8, we used the results from the clustering described above. The PICRUSt results were then analyzed using linear discriminant analysis effect size (LEfSe) to identify microbial functions that were significantly different in their abundance between groups. FishTaco was used to link taxonomic and functional shifts in the microbiome (Manor and Borenstein, 2017). The R programming environment (R Core Team, 2015) was used to generate the graphical outputs.

### Statistics

R (R Core Team, 2015) and Prism were used to perform the statistical analysis. Multiple comparisons were evaluated statistically by one-way ANOVA. Statistically significant differences were then evaluated by two-tailed Student’s *t*-test and multiple testing was corrected via false discovery rate (FDR) (Benjamini and Hochberg, 1995) estimation.

## Results

### Dietary Heme Induces Dysbiosis Similar to That Found in DSS-Treated Mice

**FIGURE 1:**
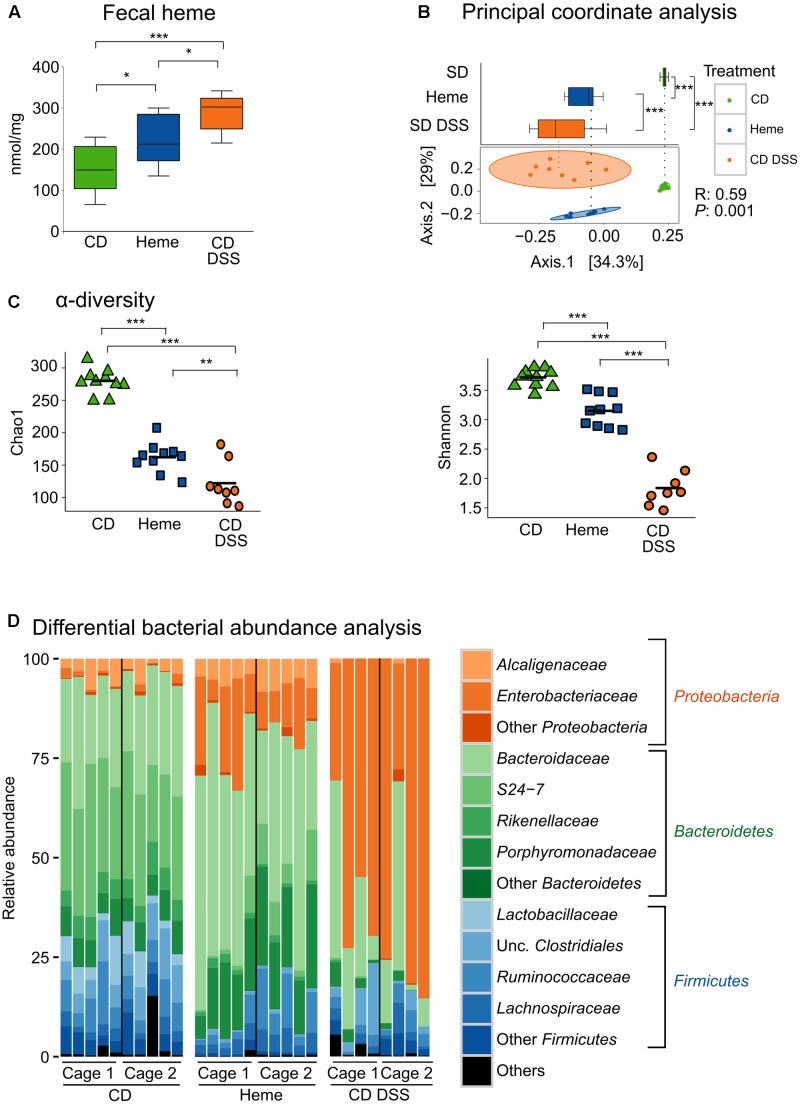
Dietary heme induces gut dysbiosis similar to that found in DSS-treated mice. Mice were fed a control diet (CD) or a heme-supplemented diet (Heme) for 4 weeks, or were fed a control diet (CD) and received either water alone, or water with DSS (CD DSS) for 10 days (*N* = 8–10 mice per group). **(A)** Fecal heme. **(B)** principal coordinate analysis with plotting of Axis.1 only (upper part) or both Axis.1 and Axis.2 (lower part). **(C)** Shannon and Chao1 measurements of α-diversity. **(D)** Differential bacterial abundance analysis. ^∗^*P* < 0.05; ^∗∗^*P* < 0.01; ^∗∗∗^*P* < 0.001. n.s., not significant. (**A,B** – upper part) Each box plot has a lower tail that extends from the minimum value to the 25th percentile; a central box that begins at the 25th percentile and ends at the 75th percentile, with a line demarcating the median; and an upper tail that extends to the maximum value. **(C)** Each symbol in both graphics represents one mouse and the line demarks the mean.

To investigate the effects of heme iron on gut microbiota composition, we analyzed microbiota in stool samples from mice fed a diet containing 50 ppm as iron-sulfate (control diet) or 50 ppm of iron as heme, and compared to mice fed with the control diet while subjected to DSS-induced colitis, which causes gastrointestinal bleeding. As shown in **Figure 1A**, stool heme levels were increased in mice fed the heme-supplemented diet and in mice subjected to DSS. PCoA (**Figure 1B**) showed that mice fed the control diet clustered separately from mice fed the heme-supplemented diet, indicating that heme iron substantially altered the composition of the gut microbiota. When analyzing α-diversity for richness (Chao1), or for both evenness and richness (Shannon), DSS-treatment showed decreased microbial diversity (**Figure 1C**), as expected (Sartor, 2008; Manichanh et al., 2012). Notably, heme dietary supplementation also significantly decreased microbial diversity, albeit to a lesser extent than DSS-treatment (**Figure 1C**).

Taxonomic composition analysis revealed that, at the phylum level, DSS-treatment resulted in increased abundance of *Proteobacteria* and reduction of *Firmicutes* and *Bacteroidetes* (**Figure 1D**). Most importantly, the changes in levels of *Proteobacteria* and *Firmicutes* were reproduced in mice fed with the heme-supplemented diet (**Figure 1D**).

**FIGURE 2:**
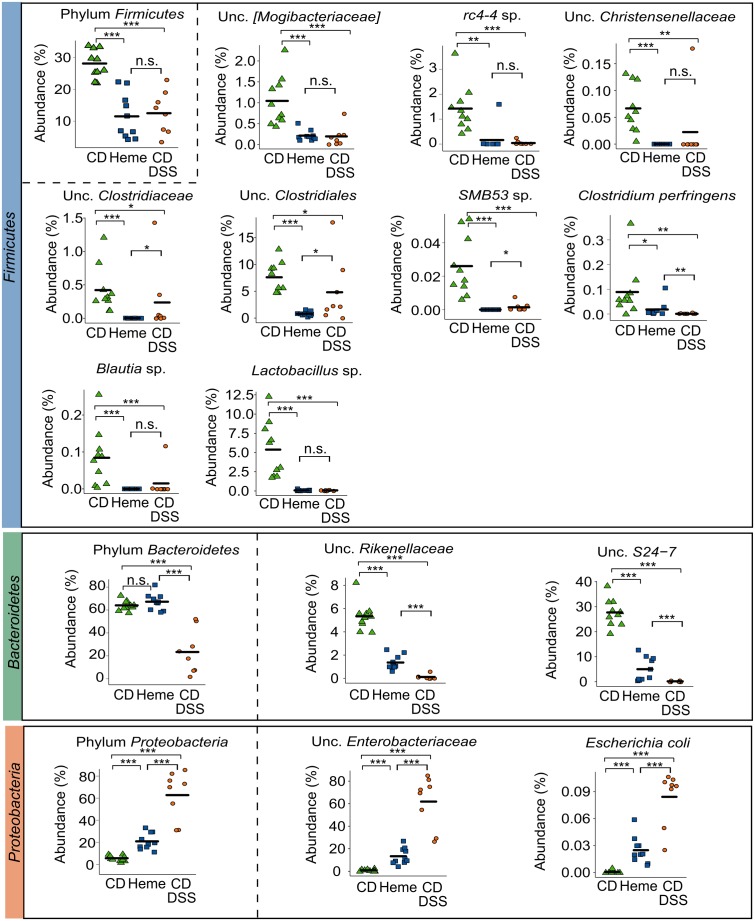
Dietary heme results in shifts in important gut bacterial species that are also modulated in DSS-treated mice. Mice were fed a control diet (CD) or a heme-supplemented diet (Heme) for 4 weeks, or were fed a control diet (CD) and received either water alone, or water with DSS (CD DSS) for 10 days (*N* = 8–10 mice per group). ^∗^*P* < 0.05; ^∗∗^*P* < 0.01; ^∗∗∗^*P* < 0.001. n.s., not significant. Each symbol represents one mouse and the line demarks the mean.

Within the phylum *Firmicutes*, and at the species level, we found that mice fed the heme-supplemented diet had similar microbial shifts as DSS-treated mice, namely a marked decrease of unclassified (unc.) [*Mogibacteriaceae*], *rc4-4* sp., unc. *Clostridiaceae*, unc. *Clostridiales*, *SMB53* sp., *Clostridium perfringens*, and *Blautia* sp. (all from the class *Clostridia*), and *Lactobacillus* sp. (class *Bacilli*) (**Figure 2**). In the phylum *Bacteroidetes*, two species were decreased in mice fed the heme-supplemented diet and in DSS-treated mice, namely unc. *Rikenellaceae* and unc. *S24-7* (**Figure 2**), whereas in the phylum *Proteobacteria*, unc. *Enterobacteriaceae* and *Escherichia coli* levels increased (**Figure 2**).

Thus, dietary heme iron induces mild dysbiosis with similar characteristics to the DSS-induced dysbiosis in mice.

### Dietary Heme Aggravates DSS-Induced Colitis and Facilitates Adenoma Formation

Previous studies have reported that, when heme iron is administered systemically, it has a protective effect on DSS-induced colitis (Zhang L. et al., 2014), an effect opposite to the one expected based on the heme-induced microbiota changes shown in **Figures 1**, **2**. Therefore, to further investigate the impact of heme iron on DSS-induced colitis, we compared colitis severity in mice fed with the diet supplemented with heme to mice fed with the control diet and receiving heme administered intraperitoneally to bypass the lumen of the gut.

**FIGURE 3:**
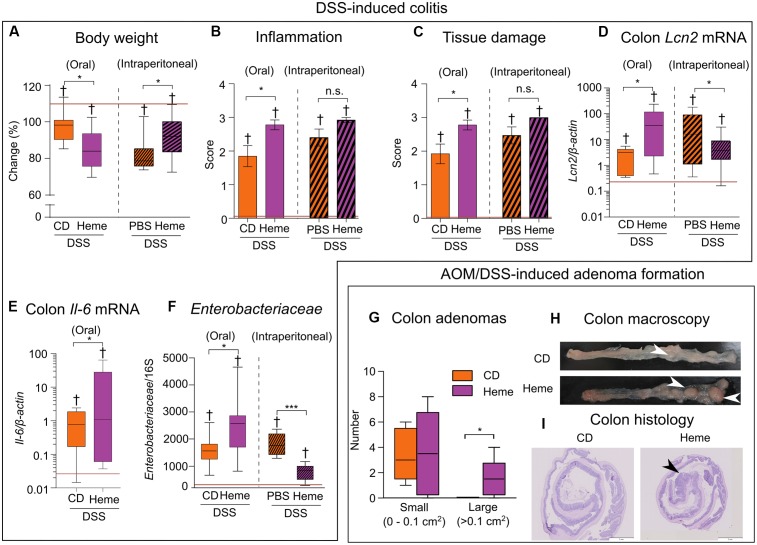
Dietary heme adversely impacts DSS-induced colitis and promotes adenoma formation. **(A–F)** *Acute colitis:* All mice received DSS in drinking water while fed a control diet (CD) versus a heme-supplemented diet (Heme) (clear bars; *N* = 7–10 mice per group) or were kept on the control diet and were injected with either phosphate-buffered saline (PBS) alone or heme solution intraperitoneally (Heme) (patterned bars; *N* = 13–15 mice). **(A)** Body weight change (final/initial body weight × 100). **(B)** Inflammation score. **(C)** Tissue damage score. **(D)** Colon *lipocalin 2* (*Lcn2*) mRNA levels. **(E)** Colon *interleukin-6* (*Il-6*) mRNA levels. **(F)** *Enterobacteriaceae* relative abundance. Red lines indicate values of mice that did not receive DSS. **(G–I)** *Azoxymethane (AOM)/DSS adenoma formation:* Mice were fed a control diet (CD) or heme-supplemented diet (Heme) (*N* = 10 mice per group). **(G)** Number of small (left) and large (right) adenomas per mouse. **(H)** Colon macroscopic appearance (white arrowheads – large adenomas). **(I)** Histological appearance of rolled colon (posterior side in the center – black arrowhead prominent adenoma). ^†^*P* < 0.001 when compared with non-DSS treated mice. ^∗^*P* < 0.05; ^∗∗∗^*P* < 0.001. n.s., not significant. **(A,D–G)** Each box plot has a lower tail that extends from the minimum value to the 25th percentile; a central box that begins at the 25th percentile and ends at the 75th percentile, with a line demarcating the median; and an upper tail that extends to the maximum value. **(B,C)** Bars represent mean ± standard error of the mean (SEM).

Compared with mice receiving the control diet, mice that received the heme-supplemented diet lost more body weight when treated with DSS (**Figure 3A**), had an increased inflammation (**Figure 3B**), and tissue damage scores (**Figure 3C**) as well as higher levels of the inflammatory markers *lipocalin 2* (*Lcn2*) (Thorsvik et al., 2017; **Figure 3D**) and *interleukin-6* (*Il-6*) (**Figure 3E**) in the gut. These changes were accompanied by an increase in *Enterobacteriaceae* levels (**Figure 3F**). In contrast, when compared to control mice receiving PBS, mice receiving heme intraperitoneally had a milder DSS-induced colitis as indicated by higher body weights (**Figure 3A**), as well as lower *Lcn2* mRNA levels (**Figure 3D**) and reduced *Enterobacteriaceae* levels (**Figure 3F**), with the inflammation (**Figure 3B**) and tissue damage (**Figure 3C**) scores remaining unaffected.

The presence of inflammation in the gut for long periods of time augments the risk for the development of colon cancer (Ekbom et al., 1990; Schetter et al., 2009; Terzic et al., 2010).We next studied the impact of dietary heme in the AOM/DSS mouse model of carcinogenesis. Using this model, we found a higher number of large adenomas in the colons of mice fed the heme-supplemented diet compared to those fed with the control diet (**Figure 3G**), as analyzed macroscopically (**Figure 3H**). Histology analysis (**Figure 3I**) revealed the adenomatous nature of the polyps.

Taken together, these results show that dietary heme, but not systemically administered heme, aggravates DSS-induced colitis and facilitates the growth of adenomas.

### Inferred Functional Profile of Mice Fed a Heme-Supplemented Diet Replicates Metabolic Shifts Found in DSS-Treated Mice

Since we found that mice subjected to the heme-supplemented diet had dysbiosis, we next investigated whether the microbiota changes would result in alterations of bacterial metabolic pathways using PICRUSt to infer the metagenomes from the 16S rRNA gene analysis.

**FIGURE 4:**
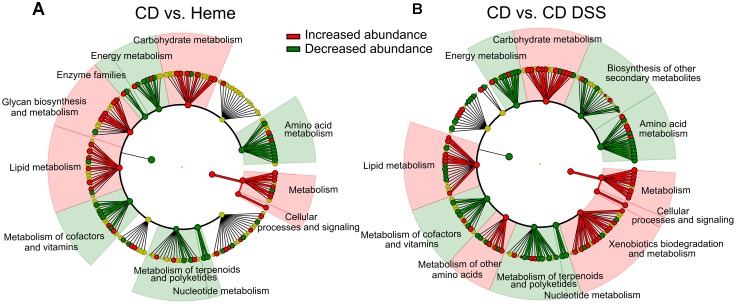
DSS and dietary heme induce similar modulation of the fecal metagenomic functional content. PICRUSt was used to predict the metagenome functional content of mice fed a control diet (CD) or a heme-supplemented diet (Heme) for 4 weeks **(A)**, or of mice fed an CD and received water alone (CD) or water with DSS (CD DSS) for 10 days **(B)** (*N* = 8–10 mice). **(A,B)** Circular dendrograms represent the KEGG functional hierarchy. The outermost circles represent individual metabolic modules and the innermost circles represent very broad functional categories. Red and green coloration denotes modules showing significant differential abundances identified with LEfSe (*P*-value cut-off of 0.05 and LDA score cut-off of 2).

Linear discriminative analysis (LDA) effect size (LEfSe) plotted in a dendogram showed that heme- and DSS-treatments resulted in similar overall metabolic profile changes when each were individually compared to untreated mice fed the control diet (**Figure 4**). The most marked changes in KEGG pathways were the increase in genes implicated in *carbohydrate* and *lipid metabolism* as well as *cellular processes and signaling*, and a decrease in *energy*, *amino acid*, and *nucleotide metabolism* genes as well as in the genes implicated in the *metabolism of terpenoids*, *polyketides and cofactors*, *and vitamins*.

**FIGURE 5:**
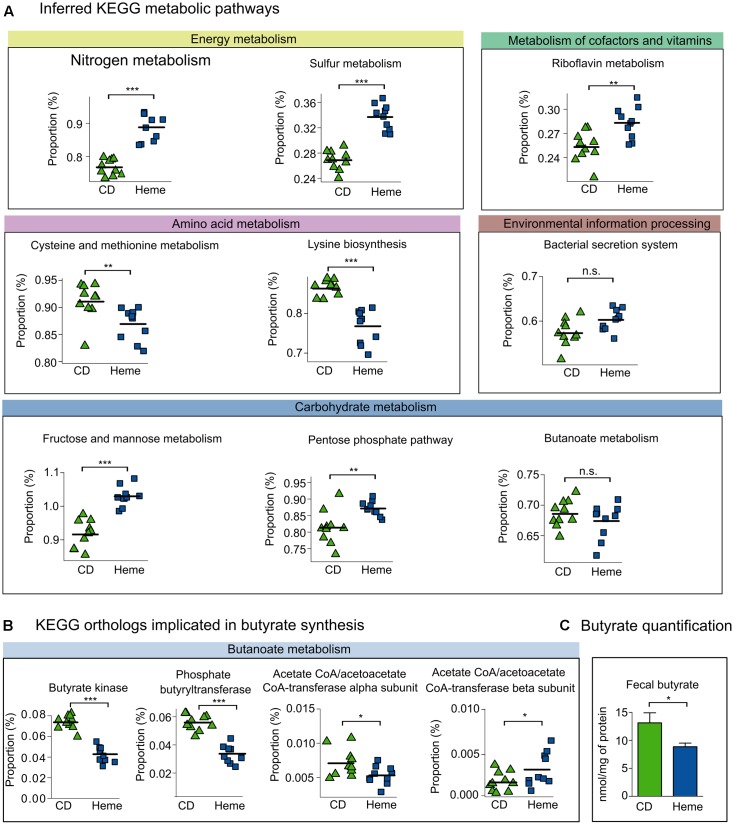
Inferred metagenome functional content of heme-fed mice replicates metabolic shifts present in IBD patients. Mice were fed a control diet (CD) or a heme-supplemented diet (Heme) for 4 weeks, or were fed a control diet (CD) and received either water alone, or water with DSS (CD DSS) for 10 days (*N* = 8–10 mice per group). **(A)** Kyoto Encyclopedia of Genes and Genomes (KEGG) pathways reported to be altered in IBD (Morgan et al., 2012). **(B)** KEGG pathways implicated in colon health. **(C)** Butyrate quantification. ^∗^*P* < 0.05; ^∗∗^*P* < 0.01; ^∗∗∗^*P* < 0.001. n.s., not significant. **(A,B)** Each symbol represents one mouse and the line demarks the mean. **(C)** Bars represent mean ± SEM.

Next, we further investigated KEGG metabolic pathways that have been reported to be specifically altered in IBD patients (Morgan et al., 2012). As shown in **Figure 5A**, we found that heme-fed mice presented with a higher number of *nitrogen* and *sulfur metabolism* genes in regards to *energy metabolism*; an elevated number of *riboflavin metabolism* genes from the *metabolism of cofactors and vitamins* pathway; and a decrease in *amino acid metabolism* genes, namely *cysteine and methionine metabolism* and *lysine biosynthesis*. *Bacterial secretion system* genes related to the *environmental information processing* pathway remained unchanged. In the *carbohydrate metabolism* pathway, we found an increase in *fructose and mannose metabolism* and *pentose phosphate pathway* genes, whereas *butanoate* (or butyrate) *metabolism* genes were not affected. Although there were no significant differences in the whole *butanoate metabolism* pathways between mice fed the heme-supplemented diet and mice fed the control diet, we did find a significant decrease in the metagenomic presence of butyrate kinase, phosphate butyryltransferase, and the α-subunit of butyryl-CoA:acetate Co-A transferase (with an increase in the β-subunit) in mice receiving the heme-supplemented diet (**Figure 5B**). We further confirmed that dietary heme diminished butyrate production by directly quantifying butyrate in fecal samples (**Figure 5C**).

Overall, these data show that dysbiosis induced by dietary heme is accompanied by several bacterial metabolic alterations.

**FIGURE 6:**
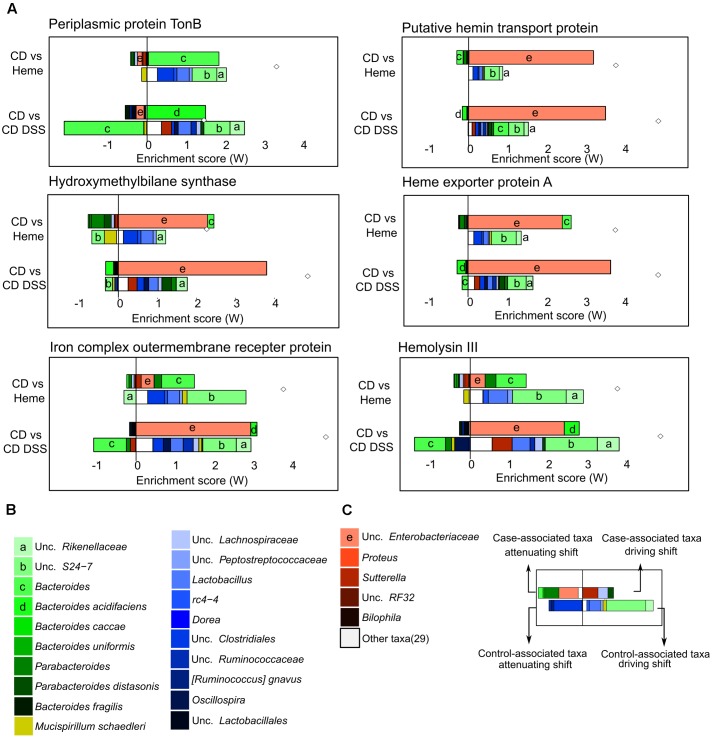
Taxonomic contributors to iron metabolism genes determined by FishTaco. Mice were fed a control diet (CD) or a heme-supplemented diet (Heme) for 4 weeks, or were fed a control diet (CD) and received either water alone, or water with DSS for 10 days (CD DSS) (*N* = 8–10 mice per group). **(A)** Taxon-level shift contribution profiles for several genes enriched by both Heme- and DSS-treatments. **(B)** Legend common to all plots. **(C)** Example explanation of a FishTaco-based taxon-level contribution profile.

Next, to further understand how heme iron may affect bacterial iron-related pathways, we curated a list of iron-related genes through KEGG database and literature searches (**Supplementary Table S2**), identifying 134 iron-related genes of which 120 were also present in the PICRUSt reference files. We then used FishTaco, a new framework for identifying taxonomic drivers of functional shifts in the microbiome (Manor and Borenstein, 2017), to investigate which bacterial species were responsible for the enrichment of identified iron-related genes triggered by dietary heme or DSS-induced colitis (**Figures 6A–C** and **Supplementary Figures S1**, **S2**). Among others, we found periplasmic TonB protein (heme uptake), a putative hemin transport protein (heme uptake), a hydroxymethylbilane synthase (heme synthesis), heme exporter protein A (heme export), iron complex outer membrane receptor protein (iron-siderophore uptake), and hemolysin III (implicated in red blood cell lysis) to be enriched in mice fed the heme supplemented diet as well as in DSS-treated mice, when compared to control mice receiving the control diet (**Figures 6A–C**). These shifts were mainly due to the presence of *Bacteroides* and unc. *Enterobacteriaceae* and the absence of unc. *S27-7*, unc. *Rikenellaceae*, and several *Firmicutes* in the mice fed the heme-supplemented diet and in DSS-treated mice. Many of the iron-related functional shifts were present in both mice fed the heme enriched diet and in DSS-treated mice (**Supplementary Figures S1**, **S2**).

## Discussion

Diet is a potent modulator of the microbial community in the gut with significant impacts on the bacterial population and microbiota health (Carmody et al., 2015). The Western diet is characterized by a high intake of fat and red meat (e.g., beef, lamb, pork) that is rich in heme. Fat has also been reported to contribute to colitis (Devkota et al., 2012; Kim et al., 2012; Gulhane et al., 2016) and colon tumor formation (Park et al., 2012). Previous studies have found that heme aggravates colitis when administered concomitantly with high fat levels (van der Logt et al., 2013), making it difficult to distinguish separate effects of heme and fat. In the present study, we show that dietary heme iron aggravates colitis and facilitates adenoma formation even when using a low-fat diet, further highlighting the importance of dietary heme in inflammation-related CRC development.

Heme has been proposed to contribute to inflammation and carcinogenesis by inducing cytotoxicity that damages the colon surface epithelium, catalyzes lipid peroxidation reactions, and produces free radicals (Ishikawa et al., 2010; Schepens et al., 2011; Ijssennagger et al., 2013; van der Logt et al., 2013), all of which are inducers of cellular damage. Our study suggests a new mechanism by which dietary heme may contribute to CRC, namely by contributing to gut dysbiosis. Heightened CRC risk has been associated with non-beneficial changes in the gut microbiota composition (Gill and Rowland, 2002; Zhan et al., 2013), reflected by an imbalance between “harmful” (e.g., *Escherichia*) and “beneficial” (e.g., *Lactobacillus*) bacteria, which influence carcinogen bioactivation and cancer risk. Patients with adenomas, compared to healthy controls, present with decreased abundance of class *Clostridia* and *[Mogibacteriaceae]*, *Christensenellaceae*, and *Clostridiaceae* families (Peters et al., 2016), which we also found decreased in the mice fed the heme-supplemented diet. Many species belonging to those taxa are important producers of butyrate (Pryde et al., 2002), a bacteria-produced metabolite with anti-inflammatory and anti-tumorigenic properties (Hague et al., 1995; Segain et al., 2000; Pryde et al., 2002; Zhang et al., 2016). The decrease in butyrate producing taxa in mice fed a heme-supplemented diet was associated with reduced gene levels of butyrate production pathways (particularly the butyrate kinase/phosphate butyryltransferase pathway) and reduced stool butyrate levels. These findings suggest that one of the mechanisms by which heme aggravates colitis and facilitates adenoma formation is by inducing dysbiosis and consequently reducing colon butyrate levels.

Our data are in agreement with previous work which used classical culturing methods and found that dietary heme increased *Enterobacteriaceae* and decreased *Lactobacilli* in rats (Schepens et al., 2011). Similarly, others found, using the Mouse Intestinal Tract Chip, that dietary heme increases the abundance of *Proteobacteria* and decreases *Firmicutes* (Ijssennagger et al., 2012).

Importantly, a low-fat diet supplemented with heme, unlike non-heme iron formulations (Constante et al., 2017), is sufficient in replicating several aspects of the dysbiosis found in IBD patients (Manichanh et al., 2006; Scanlan et al., 2006; Frank et al., 2007; Peterson et al., 2008; Walters et al., 2014; Winter and Baumler, 2014) and in the DSS mouse model of colitis (Sartor, 2008; Manichanh et al., 2012). This includes a reduction of *Firmicutes* and an increase of *Proteobacteria*, particularly *Enterobacteriaceae* (a hallmark of dysbiosis in IBD; Winter and Baumler, 2014), and an overall reduction in α-diversity. In IBD, and to a lesser extent also in CRC, gastrointestinal bleeding can further contribute to elevate luminal heme levels. Our data suggest that high luminal heme levels ensuing from gastrointestinal blood losses in IBD and CRC patients may be an important contributor to gut dysbiosis.

Heme-induced dysbiosis ultimately impacted butyrate metabolism where there are two pathways for conversion of butyrate-CoA into butyrate, a SCFA important for gut health that has been shown to have immunomodulatory properties (Inan et al., 2000; Saemann et al., 2000; Canani et al., 2011). One pathway involves a two-step reaction that uses the enzymes butyrate kinase and phosphate butyryltransferase, and the other pathway has a single-step reaction that is driven by butyryl-CoA:acetate Co-A transferase. The decrease in butyrate kinase, phosphate butyryltransferase, and the α subunit of butyryl-CoA:acetate Co-A transferase found in mice receiving the heme-supplemented diet likely contribute to the reduction in fecal butyrate levels.

There are several non-exclusive mechanisms by which heme may modulate the gut microbiota and affect bacterial metabolism. For example, luminal heme levels may favor the growth of bacteria that are capable of efficiently acquiring iron from this source. Indeed, we find an enrichment in iron metabolism genes that contribute to heme release from red blood cells (e.g., hemolysin III; Chen et al., 2004) or are responsible for heme uptake (e.g., periplasmic TonB, putative hemin transport protein; Tong and Guo, 2009) in response to elevated dietary heme. Concomitantly, bacteria may be selected for their capacity to handle heme-induced cellular toxicity when intracellular heme reaches harmful levels through heme export, sequestration, and degradation strategies (Anzaldi and Skaar, 2010). Accordingly, we found an enrichment in heme exporter proteins in the gut of mice receiving the heme-supplemented diet (e.g., heme exporter A).

Heme may additionally impact bacterial nutrient availability through its ability to reduce mucin levels (Ijssennagger et al., 2015), a source of amino acids for colon microbiota. This may explain the reduction in *amino acid metabolism*, including *cysteine and methionine metabolism* and *lysine biosynthesis* found in mice receiving the diet supplemented with heme. Finally, heme may exert a selective pressure through oxidative stress (Nath et al., 1998), resulting in the increase of the *pentose phosphate pathway* (which has NADPH as a product) and *riboflavin metabolism* genes, as both NADPH and riboflavin are important for pH and oxidative stress homeostasis (Morgan et al., 2012).

Overall these data indicate that dietary heme and DSS-induced colitis exert similar selective pressures on the gut bacterial community in regards to heme and/or iron management.

## Conclusion

**FIGURE 7:**
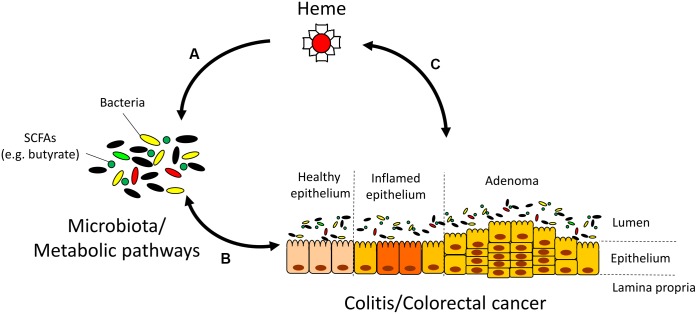
Interactions between heme, the microbiota, and colitis/colorectal cancer (CRC). **(A)** Heme alters the microbiota and its metabolic pathways that are responsible for the production of short-chain fatty acids (SCFAs). **(B)** The microbiota plays a pivotal role in inflammation and cancer development through mechanisms that include the production of SCFAs. In turn, inflammation and CRC also shape the microbiota composition (Arthur et al., 2012; Kang and Martin, 2017). **(C)** Gastrointestinal bleeding in colitis and CRC contribute to rising luminal heme levels. In turn, heme may additionally aggravate colitis and CRC development through mechanisms independent of heme-induced microbiota changes such as the induction of cytotoxicity, catalysis of lipid peroxidation reactions, and production of free radicals (Ishikawa et al., 2010; Ijssennagger et al., 2013).

Our data suggest that luminal heme, originating from dietary components or gastrointestinal bleeding directly contributes to microbiota dysbiosis found in DSS-induced colitis and in IBD patients, and may further exacerbate colitis through the modulation of the gut microbiota and its metagenomic functional composition (**Figure 7**). If heme can also be demonstrated to produce similar shifts in gut microbiota composition in humans, heme chelation or degradation using probiotics could represent a new therapeutic approach to reverse dysbiosis and prevent CRC in at risk populations.

## Author Contributions

MC contributed to the conception of the work, acquired, analyzed, and interpreted the data, and drafted and reviewed the manuscript. GF, AC, and MS-M acquired and analyzed the data, and reviewed the manuscript. MS contributed to the conception of the work and interpreted the data, and drafted and reviewed the manuscript.

## Conflict of Interest Statement

The authors declare that the research was conducted in the absence of any commercial or financial relationships that could be construed as a potential conflict of interest.

## Supplementary Material

TABLE S1 Diet compositions – detailed composition of used diets.

TABLE S2 KEGG iron-related genes – curated list of iron-related genes through Kyoto Encyclopedia of Genes and Genomes (KEGG) database and literature searches.

FIGURE S1 Taxon-level shift contribution profiles for several genes enriched by a heme-supplemented diet determined by FishTaco. Mice were fed a control diet (CD) or a heme-supplemented diet (Heme) for 4 weeks (*N* = 10 mice per group).

FIGURE S2 Taxon-level shift contribution profiles for several genes enriched by dextran sodium sulfate (DSS) determined by FishTaco. Mice were fed a control diet (CD) and received either water alone, or water with DSS (CD DSS) for 10 days (*N* = 8 mice per group).

## Abbreviations

ANOVA: analysis of variance
AOM: azoxymethane
CD: Crohn’s disease
CRC: colorectal cancer
DSS: dextran sodium sulfate
FDR: false discovery rate
IBD: inflammatory bowel disease
KEGG: Kyoto Encyclopedia of Genes and Genomes
LEfSe: linear discriminant analysis effect size
PICRUSt: phylogenetic investigation of communities by reconstruction of unobserved states.

## References

[B1] Andrews S. C. Robinson A. K. Rodriguez-Quinones F. (2003). Bacterial iron homeostasis. *FEMS Microbiol. Rev.* 27 215–237. 10.1016/S0168-6445(03)00055-X 12829269

[B2] Anzaldi L. L. Skaar E. P. (2010). Overcoming the heme paradox: heme toxicity and tolerance in bacterial pathogens. *Infect. Immun.* 78 4977–4989. 10.1128/IAI.00613-10 20679437 PMC2981329

[B3] Armstrong B. Doll R. (1975). Environmental factors and cancer incidence and mortality in different countries, with special reference to dietary practices. *Int. J. Cancer* 15 617–631. 10.1002/ijc.2910150411 1140864

[B4] Arthur J. C. Perez-Chanona E. Muhlbauer M. Tomkovich S. Uronis J. M. Fan T. J. (2012). Intestinal inflammation targets cancer-inducing activity of the microbiota. *Science* 338 120–123. 10.1126/science.1224820 22903521 PMC3645302

[B5] Becker K. W. Skaar E. P. (2014). Metal limitation and toxicity at the interface between host and pathogen. *FEMS Microbiol. Rev.* 38 1235–1249. 10.1111/1574-6976.12087 25211180 PMC4227937

[B6] Benjamini Y. Hochberg Y. (1995). Controlling the false discovery rate: a practical and powerful approach to multiple testing. *J. R. Statist. Soc. B* 57 289–300.

[B7] Canani R. B. Costanzo M. D. Leone L. Pedata M. Meli R. Calignano A. (2011). Potential beneficial effects of butyrate in intestinal and extraintestinal diseases. *World J. Gastroenterol.* 17 1519–1528. 10.3748/wjg.v17.i12 21472114 PMC3070119

[B8] Caporaso J. G. Kuczynski J. Stombaugh J. Bittinger K. Bushman F. D. Costello E. K. (2010). QIIME allows analysis of high-throughput community sequencing data. *Nat. Methods* 7 335–336. 10.1038/nmeth.f.303 20383131 PMC3156573

[B9] Carmody R. N. Gerber G. K. Luevano J. M. Jr. Gatti D. M. Somes L. (2015). Diet dominates host genotype in shaping the murine gut microbiota. *Cell Host Microbe* 17 72–84. 10.1016/j.chom.2014.11.010 25532804 PMC4297240

[B10] Cescau S. Cwerman H. Letoffe S. Delepelaire P. Wandersman C. Biville F. (2007). Heme acquisition by hemophores. *Biometals* 20 603–613. 10.1007/s10534-006-9050-y 17268821

[B11] Chassaing B. Aitken J. D. Malleshappa M. Vijay-Kumar M. (2014). Dextran sulfate sodium (DSS)-induced colitis in mice. *Curr. Protoc. Immunol.* 104 1525.1–15.25.14. 10.1002/0471142735.im1525s104 PMC3980572 24510619

[B12] Chen Y. C. Chang M. C. Chuang Y. C. Jeang C. L. (2004). Characterization and virulence of hemolysin III from *Vibrio vulnificus*. *Curr. Microbiol.* 49 175–179. 10.1007/s00284-004-4288-5 15386100

[B13] Constante M. Fragoso G. Lupien-Meilleur J. Calve A. Santos M. M. (2017). Iron supplements modulate colon microbiota composition and potentiate the protective effects of probiotics in dextran sodium sulfate-induced colitis. *Inflamm. Bowel Dis.* 23 753–766. 10.1097/MIB.0000000000001089 28368910

[B14] De Robertis M. Massi E. Poeta M. L. Carotti S. Morini S. Cecchetelli L. (2011). The AOM/DSS murine model for the study of colon carcinogenesis: from pathways to diagnosis and therapy studies. *J. Carcinog.* 10:9 10.4103/1477-3163.78279 PMC3072657 21483655

[B15] De Stefani E. Boffetta P. Ronco A. L. Deneo-Pellegrini H. Correa P. Acosta G. (2012). Processed meat consumption and risk of cancer: a multisite case-control study in Uruguay. *Br. J. Cancer* 107 1584–1588. 10.1038/bjc.2012.433 23011480 PMC3493769

[B16] Devkota S. Wang Y. Musch M. W. Leone V. Fehlner-Peach H. Nadimpalli A. (2012). Dietary-fat-induced taurocholic acid promotes pathobiont expansion and colitis in Il10-/- mice. *Nature* 487 104–108. 10.1038/nature11225 22722865 PMC3393783

[B17] Diaz-Ochoa V. E. Jellbauer S. Klaus S. Raffatellu M. (2014). Transition metal ions at the crossroads of mucosal immunity and microbial pathogenesis. *Front. Cell Infect. Microbiol.* 4:2 10.3389/fcimb.2014.00002 PMC3900919 24478990

[B18] Ekbom A. Helmick C. Zack M. Adami H. O. (1990). Ulcerative colitis and colorectal cancer. A population-based study. *N. Engl. J. Med.* 323 1228–1233. 10.1056/NEJM199011013231802 2215606

[B19] Frank D. N. St Amand A. L. Feldman R. A. Boedeker E. C. Harpaz N. Pace N. R. (2007). Molecular-phylogenetic characterization of microbial community imbalances in human inflammatory bowel diseases. *Proc. Natl. Acad. Sci. U.S.A.* 104 13780–13785. 10.1073/pnas.0706625104 17699621 PMC1959459

[B20] Gill C. I. Rowland I. R. (2002). Diet and cancer: assessing the risk. *Br. J. Nutr.* 88(Suppl. 1), S73–S87. 10.1079/BJN2002632 12215186

[B21] Gulhane M. Murray L. Lourie R. Tong H. Sheng Y. H. Wang R. (2016). High fat diets induce colonic epithelial cell stress and inflammation that is reversed by IL-22. *Sci. Rep.* 6:28990 10.1038/srep28990 PMC4924095 27350069

[B22] Hague A. Elder D. J. Hicks D. J. Paraskeva C. (1995). Apoptosis in colorectal tumour cells: induction by the short chain fatty acids butyrate, propionate and acetate and by the bile salt deoxycholate. *Int. J. Cancer* 60 400–406. 10.1002/ijc.2910600322 7829251

[B23] Han J. Lin K. Sequeira C. Borchers C. H. (2015). An isotope-labeled chemical derivatization method for the quantitation of short-chain fatty acids in human feces by liquid chromatography-tandem mass spectrometry. *Anal. Chim. Acta* 854 86–94. 10.1016/j.aca.2014.11.015 25479871

[B24] Huang H. Constante M. Layoun A. Santos M. M. (2009). Contribution of STAT3 and SMAD4 pathways to the regulation of hepcidin by opposing stimuli. *Blood* 113 3593–3599. 10.1182/blood-2008-08-173641 19204324 PMC2891008

[B25] Ijssennagger N. Belzer C. Hooiveld G. J. Dekker J. van Mil S. W. Muller M. (2015). Gut microbiota facilitates dietary heme-induced epithelial hyperproliferation by opening the mucus barrier in colon. *Proc. Natl. Acad. Sci. U.S.A.* 112 10038–10043. 10.1073/pnas.1507645112 26216954 PMC4538683

[B26] Ijssennagger N. Derrien M. van Doorn G. M. Rijnierse A. van den Bogert B. Muller M. (2012). Dietary heme alters microbiota and mucosa of mouse colon without functional changes in host-microbe cross-talk. *PLOS ONE* 7:e49868 10.1371/journal.pone.0049868 PMC3519815 23239972

[B27] Ijssennagger N. Rijnierse A. de Wit N. J. Boekschoten M. V. Dekker J. Schonewille A. (2013). Dietary heme induces acute oxidative stress, but delayed cytotoxicity and compensatory hyperproliferation in mouse colon. *Carcinogenesis* 34 1628–1635. 10.1093/carcin/bgt084 23455377

[B28] Inan M. S. Rasoulpour R. J. Yin L. Hubbard A. K. Rosenberg D. W. Giardina C. (2000). The luminal short-chain fatty acid butyrate modulates NF-kappaB activity in a human colonic epithelial cell line. *Gastroenterology* 118 724–734. 10.1016/S0016-5085(00)70142-9 10734024

[B29] Ishikawa S. Tamaki S. Ohata M. Arihara K. Itoh M. (2010). Heme induces DNA damage and hyperproliferation of colonic epithelial cells via hydrogen peroxide produced by heme oxygenase: a possible mechanism of heme-induced colon cancer. *Mol. Nutr. Food Res.* 54 1182–1191. 10.1002/mnfr.200900348 20112302

[B30] Jia Q. Lupton J. R. Smith R. Weeks B. R. Callaway E. Davidson L. A. (2008). Reduced colitis-associated colon cancer in Fat-1 (n-3 fatty acid desaturase) transgenic mice. *Cancer Res.* 68 3985–3991. 10.1158/0008-5472.CAN-07-6251 18483285 PMC2648804

[B31] Jowett S. L. Seal C. J. Pearce M. S. Phillips E. Gregory W. Barton J. R. (2004). Influence of dietary factors on the clinical course of ulcerative colitis: a prospective cohort study. *Gut* 53 1479–1484. 10.1136/gut.2003.024828 15361498 PMC1774231

[B32] Kamada N. Chen G. Y. Inohara N. Nunez G. (2013). Control of pathogens and pathobionts by the gut microbiota. *Nat. Immunol.* 14 685–690. 10.1038/ni.2608 23778796 PMC4083503

[B33] Kanehisa M. Goto S. (2000). KEGG: kyoto encyclopedia of genes and genomes. *Nucleic Acids Res.* 28 27–30. 10.1093/nar/28.1.27 10592173 PMC102409

[B34] Kang M. Martin A. (2017). Microbiome and colorectal cancer: unraveling host-microbiota interactions in colitis-associated colorectal cancer development. *Semin. Immunol.* 10.1016/j.smim.2017.04.003 [Epub ahead of print]. 28465070

[B35] Kim E. Coelho D. Blachier F. O. (2013). Review of the association between meat consumption and risk of colorectal cancer. *Nutr. Res.* 33 983–994. 10.1016/j.nutres.2013.07.018 24267037

[B36] Kim E. R. Chang D. K. (2014). Colorectal cancer in inflammatory bowel disease: the risk, pathogenesis, prevention and diagnosis. *World J. Gastroenterol.* 20 9872–9881. 10.3748/wjg.v20.i29.9872 25110418 PMC4123369

[B37] Kim K. A. Gu W. Lee I. A. Joh E. H. Kim D. H. (2012). High fat diet-induced gut microbiota exacerbates inflammation and obesity in mice via the TLR4 signaling pathway. *PLOS ONE* 7:e47713 10.1371/journal.pone.0047713 PMC3473013 23091640

[B38] Langille M. G. Zaneveld J. Caporaso J. G. McDonald D. Knights D. Reyes J. A. (2013). Predictive functional profiling of microbial communities using 16S rRNA marker gene sequences. *Nat. Biotechnol.* 31 814–821. 10.1038/nbt.2676 23975157 PMC3819121

[B39] Larsson S. C. Wolk A. (2006). Meat consumption and risk of colorectal cancer: a meta-analysis of prospective studies. *Int. J. Cancer* 119 2657–2664. 10.1002/ijc.22170 16991129

[B40] Layoun A. Santos M. M. (2012). Bacterial cell wall constituents induce hepcidin expression in macrophages through MyD88 signaling. *Inflammation* 35 1500–1506. 10.1007/s10753-012-9463-4 22544439

[B41] Lozupone C. Knight R. (2005). UniFrac: a new phylogenetic method for comparing microbial communities. *Appl. Environ. Microbiol.* 71 8228–8235. 10.1128/AEM.71.12.8228-8235.2005 16332807 PMC1317376

[B42] Makui H. Soares R. J. Jiang W. Constante M. Santos M. M. (2005). Contribution of Hfe expression in macrophages to the regulation of hepatic hepcidin levels and iron loading. *Blood* 106 2189–2195. 10.1182/blood-2005-02-0629 15914561 PMC2891009

[B43] Manichanh C. Borruel N. Casellas F. Guarner F. (2012). The gut microbiota in IBD. *Nat. Rev. Gastroenterol. Hepatol.* 9 599–608. 10.1038/nrgastro.2012.152 22907164

[B44] Manichanh C. Rigottier-Gois L. Bonnaud E. Gloux K. Pelletier E. Frangeul L. (2006). Reduced diversity of faecal microbiota in Crohn’s disease revealed by a metagenomic approach. *Gut* 55 205–211. 10.1136/gut.2005.073817 16188921 PMC1856500

[B45] Manor O. Borenstein E. (2017). Systematic characterization and analysis of the taxonomic drivers of functional shifts in the human microbiome. *Cell Host Microbe* 21 254–267. 10.1016/j.chom.2016.12.014 28111203 PMC5316541

[B46] McMurdie P. J. Holmes S. (2012). Phyloseq: a bioconductor package for handling and analysis of high-throughput phylogenetic sequence data. *Pac. Symp. Biocomput.* 17 235–246. PMC3357092 22174279

[B47] Morgan X. C. Tickle T. L. Sokol H. Gevers D. Devaney K. L. Ward D. V. (2012). Dysfunction of the intestinal microbiome in inflammatory bowel disease and treatment. *Genome Biol.* 13:R79 10.1186/gb-2012-13-9-r79 PMC3506950 23013615

[B48] Nath K. A. Grande J. P. Croatt A. J. Likely S. Hebbel R. P. Enright H. (1998). Intracellular targets in heme protein-induced renal injury. *Kidney Int.* 53 100–111. 10.1046/j.1523-1755.1998.00731.x 9453005

[B49] Norat T. Lukanova A. Ferrari P. Riboli E. (2002). Meat consumption and colorectal cancer risk: dose-response meta-analysis of epidemiological studies. *Int. J. Cancer* 98 241–256. 10.1002/ijc.10126 11857415

[B50] Oksanen J. Blanchet F. G. Kindt R. Legendre P. Minchin P. R. O’Hara R. B. (2015). *vegan: Community Ecology Package. R package Version 2.3-2*. Available at: https://CRAN.R-project.org/package=vegan

[B51] Park S. Y. Kim J. S. Seo Y. R. Sung M. K. (2012). Effects of diet-induced obesity on colitis-associated colon tumor formation in A/J mice. *Int. J. Obes.* 36 273–280. 10.1038/ijo.2011.83 21544082

[B52] Peters B. A. Dominianni C. Shapiro J. A. Church T. R. Wu J. Miller G. (2016). The gut microbiota in conventional and serrated precursors of colorectal cancer. *Microbiome* 4:69 10.1186/s40168-016-0218-6 PMC5203720 28038683

[B53] Peterson D. A. Frank D. N. Pace N. R. Gordon J. I. (2008). Metagenomic approaches for defining the pathogenesis of inflammatory bowel diseases. *Cell Host Microbe* 3 417–427. 10.1016/j.chom.2008.05.001 18541218 PMC2872787

[B54] Pryde S. E. Duncan S. H. Hold G. L. Stewart C. S. Flint H. J. (2002). The microbiology of butyrate formation in the human colon. *FEMS Microbiol. Lett.* 217 133–139. 10.1111/j.1574-6968.2002.tb11467.x 12480096

[B55] R Core Team. (2015). *R: A Language and Environment for Statistical Computing*. Vienna: R Foundation for Statistical Computing.

[B56] Reniere M. L. Torres V. J. Skaar E. P. (2007). Intracellular metalloporphyrin metabolism in *Staphylococcus aureus*. *Biometals* 20 333–345. 10.1007/s10534-006-9032-0 17387580

[B57] Saemann M. D. Bohmig G. A. Osterreicher C. H. Burtscher H. Parolini O. Diakos C. (2000). Anti-inflammatory effects of sodium butyrate on human monocytes: potent inhibition of IL-12 and up-regulation of IL-10 production. *FASEB J.* 14 2380–2382. 10.1096/fj.00-0359fje 11024006

[B58] Sandhu M. S. White I. R. McPherson K. (2001). Systematic review of the prospective cohort studies on meat consumption and colorectal cancer risk: a meta-analytical approach. *Cancer Epidemiol. Biomarkers Prev.* 10 439–446. 11352852

[B59] Sartor R. B. (2008). Microbial influences in inflammatory bowel diseases. *Gastroenterology* 134 577–594. 10.1053/j.gastro.2007.11.059 18242222

[B60] Scanlan P. D. Shanahan F. O’Mahony C. Marchesi J. R. (2006). Culture-independent analyses of temporal variation of the dominant fecal microbiota and targeted bacterial subgroups in Crohn’s disease. *J. Clin. Microbiol.* 44 3980–3988. 10.1128/JCM.00312-06 16988018 PMC1698357

[B61] Schepens M. A. Vink C. Schonewille A. J. Dijkstra G. van der Meer R. Bovee-Oudenhoven I. M. (2011). Dietary heme adversely affects experimental colitis in rats, despite heat-shock protein induction. *Nutrition* 27 590–597. 10.1016/j.nut.2010.05.002 20705428

[B62] Schetter A. J. Nguyen G. H. Bowman E. D. Mathe E. A. Yuen S. T. Hawkes J. E. (2009). Association of inflammation-related and microRNA gene expression with cancer-specific mortality of colon adenocarcinoma. *Clin. Cancer Res.* 15 5878–5887. 10.1158/1078-0432.CCR-09-0627 19737943 PMC2745503

[B63] Segain J. P. Raingeard de la Bletiere D. Bourreille A. Leray V. Gervois N. Rosales C. (2000). Butyrate inhibits inflammatory responses through NFkappaB inhibition: implications for Crohn’s disease. *Gut* 47 397–403. 10.1136/gut.47.3.397 10940278 PMC1728045

[B64] Sinha R. Cross A. J. Graubard B. I. Leitzmann M. F. Schatzkin A. (2009). Meat intake and mortality: a prospective study of over half a million people. *Arch. Intern. Med.* 169 562–571. 10.1001/archinternmed.2009.6 19307518 PMC2803089

[B65] Terzic J. Grivennikov S. Karin E. Karin M. (2010). Inflammation and colon cancer. *Gastroenterology* 138 2101–2114. 10.1053/j.gastro.2010.01.058 20420949

[B66] Thorsvik S. Damas J. K. Granlund A. V. Flo T. H. Bergh K. Ostvik A. E. (2017). Fecal neutrophil gelatinase-associated lipocalin as a biomarker for inflammatory bowel disease. *J. Gastroenterol. Hepatol.* 32 128–135. 10.1111/jgh.13598 27640344

[B67] Tong Y. Guo M. (2009). Bacterial heme-transport proteins and their heme-coordination modes. *Arch. Biochem. Biophys.* 481 1–15. 10.1016/j.abb.2008.10.013 18977196 PMC2683585

[B68] van den Berg J. W. Koole-Lesuis R. Edixhoven-Bosdijk A. Brouwers N. (1988). Automating the quantification of heme in feces. *Clin. Chem.* 34 2125–2126. 3168229

[B69] van der Logt E. M. Blokzijl T. van der Meer R. Faber K. N. Dijkstra G. (2013). Westernized high-fat diet accelerates weight loss in dextran sulfate sodium-induced colitis in mice, which is further aggravated by supplementation of heme. *J. Nutr. Biochem.* 24 1159–1165. 10.1016/j.jnutbio.2012.09.001 23246033

[B70] Walters W. A. Xu Z. Knight R. (2014). Meta-analyses of human gut microbes associated with obesity and IBD. *FEBS Lett.* 588 4223–4233. 10.1016/j.febslet.2014.09.039 25307765 PMC5050012

[B71] Wandersman C. Delepelaire P. (2004). Bacterial iron sources: from siderophores to hemophores. *Annu. Rev. Microbiol.* 58 611–647. 10.1146/annurev.micro.58.030603.123811 15487950

[B72] Weinberg E. D. (1977). Infection and iron metabolism. *Am. J. Clin. Nutr.* 30 1485–1490. 409273 10.1093/ajcn/30.9.1485

[B73] Weinberg E. D. (2009). Iron availability and infection. *Biochim. Biophys. Acta* 1790 600–605. 10.1016/j.bbagen.2008.07.002 18675317

[B74] Wilks A. Burkhard K. A. (2007). Heme and virulence: how bacterial pathogens regulate, transport and utilize heme. *Nat. Prod. Rep.* 24 511–522. 10.1039/b604193k 17534527

[B75] Winter S. E. Baumler A. J. (2014). Dysbiosis in the inflamed intestine: chance favors the prepared microbe. *Gut Microbes* 5 71–73. 10.4161/gmic.27129 24637596 PMC4049941

[B76] Zhan Y. Chen P.-J. Sadler W. D. Wang F. Poe S. Nunez G. (2013). Gut microbiota protects against gastrointestinal tumorigenesis caused by epithelial injury. *Cancer Res.* 73 7199–7210. 10.1158/0008-5472.can-13-0827 24165160 PMC3883499

[B77] Zhang J. Kobert K. Flouri T. Stamatakis A. (2014). PEAR: a fast and accurate Illumina Paired-End reAd mergeR. *Bioinformatics* 30 614–620. 10.1093/bioinformatics/btt593 24142950 PMC3933873

[B78] Zhang L. Zhang Y. Zhong W. Di C. Lin X. Xia Z. (2014). Heme oxygenase-1 ameliorates dextran sulfate sodium-induced acute murine colitis by regulating Th17/Treg cell balance. *J. Biol. Chem.* 289 26847–26858. 10.1074/jbc.M114.590554 25112868 PMC4175326

[B79] Zhang T. Ding C. Zhao M. Dai X. Yang J. Li Y. (2016). Sodium butyrate reduces colitogenic immunoglobulin A-coated bacteria and modifies the composition of microbiota in IL-10 deficient mice. *Nutrients* 8:728 10.3390/nu8120728 PMC5188405 27886121

