# Supplementary material for: Dietary Heme Induces Gut Dysbiosis, Aggravates Colitis, and Potentiates the Development of Adenomas in Mice
Source: Front Microbiol. 2017 Sep 21;8:1809. doi: 10.3389/fmicb.2017.01809 (PMC5613120; doi:10.3389/fmicb.2017.01809)
Supplement: TABLE S1 — Diet compositions – detailed composition of used diets. [file Table_1.PDF]

Control diet (TD.120515)	g/kg
Casein, low Cu & Fe	200.0
DL-Methionine	3.0
Sucrose	551.74
Corn Starch	150.0
Corn Oil	50.0
Mineral Mix, Fe Deficient (81062)	35.0
Ferrous Sulfate, heptahydrate	0.25
Vitamin Mix, Teklad (40060)	10.0
Ethoxyquin, antioxidant	0.01

Heme-supplemented (TD.120516)	g/kg
Casein, low Cu & Fe	200.0
DL-Methionine	3.0
Sucrose	551.4
Corn Starch	150.0
Corn Oil	50.0
Mineral Mix, Fe Deficient (81062)	35.0
Hemin	0.59
Vitamin Mix, Teklad (40060)	10.0
Ethoxyquin, antioxidant	0.01

Control diet (TD.140855)	g/kg
2018, Teklad Global 18% Protein Rodent Diet	500.0
Casein, low Cu & Fe	100.0
DL-Methionine	1.5
Sucrose	275.87
Corn Starch	75.0
Corn Oil	25.0
Mineral Mix, Fe Deficient (81062)	17.5
Ferrous Sulfate, heptahydrate	0.125
Vitamin Mix, Teklad (40060)	5.0
Ethoxyquin, antioxidant	0.005

Heme-supplemented (TD.140856)	g/kg
2018, Teklad Global 18% Protein Rodent Diet	500.0
Casein, low Cu & Fe	100.0
DL-Methionine	1.5
Sucrose	275.7
Corn Starch	75.0
Corn Oil	25.0
Mineral Mix, Fe Deficient (81062)	17.5
Hemin	0.295
Vitamin Mix, Teklad (40060)	5.0
Ethoxyquin, antioxidant	0.005
