# Supplementary material for: Dietary Heme Induces Gut Dysbiosis, Aggravates Colitis, and Potentiates the Development of Adenomas in Mice
Source: Front Microbiol. 2017 Sep 21;8:1809. doi: 10.3389/fmicb.2017.01809 (PMC5613120; doi:10.3389/fmicb.2017.01809)
Supplement: TABLE S2 — KEGG iron-related genes – curated list of iron-related genes through Kyoto Encyclopedia of Genes and Genomes (KEGG) database and literature searches. [file Table_2.PDF]

Group	Gene	Kegg #
Heme systems	heme acquisition protein HasR	K16152
	hemoglobin/transferrin/lactoferrin receptor protein	K16087
	HPHBR; haptoglobin-hemoglobin receptor	K14478
	HBB; hemoglobin subunit beta	K13823
	HBA; hemoglobin subunit alpha	K13822
	heme acquisition protein HasA	K12545
	hemolysin E	K11139
	hemolysin III	K11068
	multidrug/hemolysin transport system permease protein	K11051
	multidrug/hemolysin transport system ATP-binding protein	K11050
	delta-hemolysin	K11039
	leukocidin/hemolysin toxin family protein	K11038
	hemolysin BL lytic component L1	K11037
	hemolysin BL lytic component L2	K11036
	hemolysin BL binding component	K11035
	hemolysin II	K11032
	structural toxin protein (hemagglutinin/hemolysin) RtxA	K11022
	thermostable direct hemolysin 1/2	K11019
	thermolabile hemolysin	K11018
	hemolysin activation/secretion protein??	K11017
	hemolysin	K11016
	hemolysin A	K11005
	hemolysin D	K11003
	hemolysin	K10948
	heme-transporting ATPase [EC:3.6.3.41]	K10834
	hemin transport system ATP-binding protein [EC:3.6.3.-]	K09814
	Heme efflux system permease HrtB	K09813
	hemolysin activation/secretion protein	K07326
	chromate transport protein	K07240
	putative hemin transport protein	K07225
	hemerythrin	K07216
	heme oxygenase	K07215
	transmembrane sensor	K07165
	hemoglobin	K06886
	putative hemolysin	K06442
	putative hemolysin	K03699
	protoheme IX farnesyltransferase [EC:2.5.1.-]	K02301
	COX10; protoheme IX farnesyltransferase [EC:2.5.1.-]	K02257
	heme exporter protein D	K02196
	heme exporter protein C	K02195
	heme exporter protein B	K02194
	heme exporter protein A [EC:3.6.3.41]	K02193
	iron complex outermembrane recepter protein	K02014
	E4.4.1.17; cytochrome c heme-lyase [EC:4.4.1.17]	K01764
	hydroxymethylbilane synthase	K01749
	porphobilinogen synthase	K01698
	hemE, UROD; uroporphyrinogen decarboxylase [EC:4.1.1.37]	K01599
	heme oxygenase [EC:1.14.99.3]	K00510
	cysI; sulfite reductase (NADPH) hemoprotein beta-component [EC:1.8.1.2]	K00381
	coproporphyrinogen III oxidase	K00228
	hemoglobin/transferrin/lactoferrin receptor protein	K16087

Group	Gene	Kegg #
Elemental iron	iron-chelate-transporting ATPase [EC:3.6.3.34]	K10829
	Fe(3+) dicitrate transport protein	K16091
	iron(III) transport system substrate-binding protein	K02012
	iron(III) transport system permease protein	K02011
	iron(III) transport system ATP-binding protein [EC:3.6.3.30]	K02010
	ferric-chelate reductase [NAD(P)H]	K18915
	SLC40A1, FPN1; solute carrier family 40 (iron-regulated transporter), member 1	K14685
	ferrous-iron efflux pump FieF	K13283
	ferric iron reductase protein FhuF	K13255
	troB, mntB, znuC; manganese/zinc/iron transport system ATP- binding protein	K11710
	manganese/zinc/iron transport system permease protein	K11709
	manganese/zinc/iron transport system permease protein	K11708
	manganese/zinc/iron transport system substrate-binding protein	K11707
	iron/zinc/copper transport system ATP-binding protein	K11706
	iron/zinc/copper transport system permease protein	K11705
	iron/zinc/copper transport system substrate-binding protein	K11704
	manganese/iron transport system ATP-binding protein	K11607
	manganese/iron transport system permease protein	K11606
	manganese/iron transport system permease protein	K11605
	manganese/iron transport system substrate-binding protein	K11604
	putative ferrous iron transport protein C	K10123
	manganese/iron transport system ATP-binding protein	K09820
	manganese/iron transport system permease protein	K09819
	manganese/iron transport system substrate-binding protein	K09818
	ferrous iron transport protein C	K07490
	high-affinity iron transporter	K07243
	zinc transporter, ZIP family	K07238
	ferric-chelate reductase (NADPH)	K07229
	TFRC, CD71; transferrin receptor	K06503
	magnesium transporter	K06213
	ferrous iron transport protein B	K04759
	ferrous iron transport protein A	K04758
	iron complex transport system substrate-binding protein	K02016
	iron complex transport system permease protein	K02015
	iron complex transport system ATP-binding protein [EC:3.6.3.34]	K02013
	ferric-chelate reductase	K00521

Group	Gene	Kegg #
Siderophore systems	fepA, pfeA, iroN, pirA; ferric enterobactin receptor	K19611
	vitamin B12 transporter	K16092
	catecholate siderophore receptor	K16090
	outer membrane receptor for ferrienterochelin and colicins	K16089
	outer-membrane receptor for ferric coprogen and ferric-rhodotorulic acid	K16088
	pesticin/yersiniabactin receptor	K15721
	inner membrane transporter RhtA	K11939
	MFS transporter, ENTS family, enterobactin (siderophore) exporter	K08225
	enterochelin esterase and related enzymes	K07214
	yersiniabactin synthetase, thioesterase component	K05374
	mycobactin lysine-N-oxygenase	K04793
	mycobactin peptide synthetase MbtF	K04792
	mycobactin polyketide synthetase MbtD	K04791
	mycobactin polyketide synthetase MbtC	K04790
	mycobactin peptide synthetase MbtE	K04789
	mycobactin phenyloxazoline synthetase	K04788
	mycobactin salicyl-AMP ligase [EC:6.3.2.-]	K04787
	yersiniabactin nonribosomal peptide/polyketide synthase	K04786
	yersiniabactin synthetase, thiazolinyl reductase component	K04785
	yersiniabactin nonribosomal peptide synthetase	K04784
	yersiniabactin salicyl-AMP ligase [EC:6.3.2.-]	K04783
	vibriobactin synthetase	K04778
	vibriobactin-specific isochorismatase [EC:3.3.2.1]	K04777
	vibriobactin-specific 2,3-dihydroxybenzoate-AMP ligase [EC:2.7.7.58]	K04776
	aerobactin synthetase subunit beta [EC:6.3.2.27]	K03895
	aerobactin synthetase subunit alpha [EC:6.3.2.27]	K03894
	enterobactin synthetase component F [EC:2.7.7.-]	K02364
	enterobactin 2,3-dihydroxybenzoate-AMP ligase / S-dihydroxybenzoyltransferase [EC:2.7	K02363
	enterobactin synthetase component D [EC:2.7.8.-]	K02362
	enterobactin isochorismatase [EC:3.3.2.1]	K01252
	small regulatory RNA RyhB	K18507
Iron storage and regulation	TF; transferrin	K14736
	Rrf2 family transcriptional regulator, iron-responsive regulator	K13772
	FTL; ferritin light chain	K13625
	DtxR family transcriptional regulator, manganese transport regulator	K11924
	Fur family transcriptional regulator, iron response regulator	K09826
	periplasmic iron binding protein	K07230
	flavodoxin I	K03839
	periplasmic protein TonB	K03832
	Fur family transcriptional regulator, ferric uptake regulator	K03711
	DtxR family transcriptional regulator, Mn-dependent transcriptional regulator	K03709
	bacterioferritin	K03594
	biopolymer transport protein ExbB	K03561
	biopolymer transport protein ExbD	K03559
	ferritin-like protein 2	K02255
	ferritin [EC:1.16.3.1]	K02217
	bacterioferritin-associated ferredoxin	K02192
	ferritin heavy chain [EC:1.16.3.1]	K00522
