# Supplementary material for: Dietary Heme Induces Gut Dysbiosis, Aggravates Colitis, and Potentiates the Development of Adenomas in Mice
Source: Front Microbiol. 2017 Sep 21;8:1809. doi: 10.3389/fmicb.2017.01809 (PMC5613120; doi:10.3389/fmicb.2017.01809)
Supplement: FIGURE S1 — Taxon-level shift contribution profiles for several genes enriched by a heme-supplemented diet determined by FishTaco. Mice were fed a control diet (CD) or a heme-supplemented diet (Heme) for 4 weeks (N = 10 mice per group). [file Image_1.PDF]

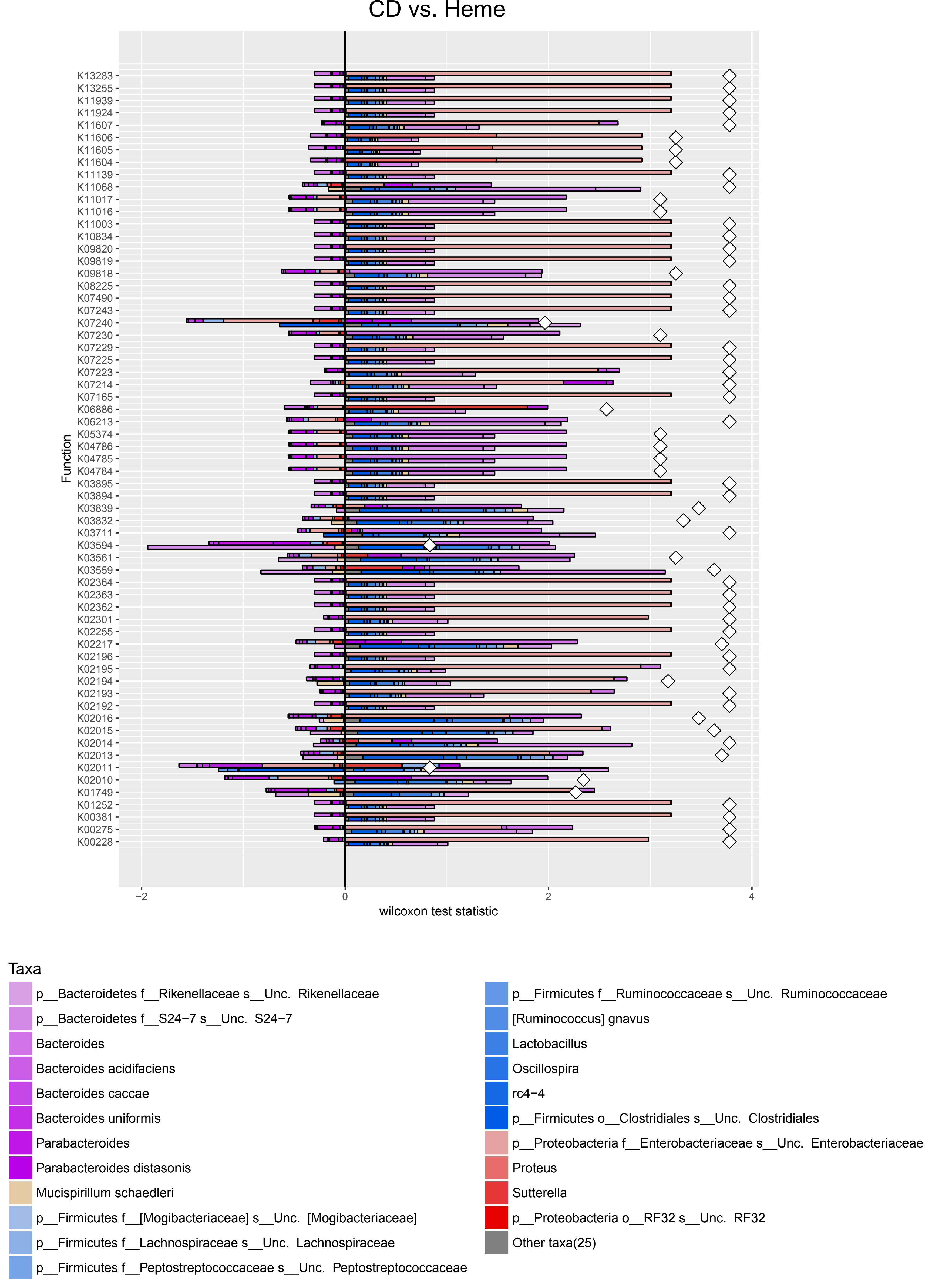

CD vs. Heme
Function
K13283
K13255
K11939
K11924
K11607
K11606
K11605
K11604
K11139
K11068
K11017
K11016
K11003
K10834
K09820
K09819
K09818
K08225
K07490
K07243
K07240
K07230
K07229
K07225
K07223
K07214
K07165
K06886
K06213
K05374
K04786
K04785
K04784
K03895
K03894
K03839
K03832
K03711
K03594
K03561
K03559
K02364
K02363
K02362
K02301
K02255
K02217
K02196
K02195
K02194
K02193
K02192
K02016
K02015
K02014
K02013
K02011
K02010
K01749
K01252
K00381
K00275
K00228
−2
0
2
4
wilcoxon test statistic
Taxa
p__Bacteroidetes f__Rikenellaceae s__Unc. Rikenellaceae
p__Bacteroidetes f__S24−7 s__Unc. S24−7
Bacteroides
Bacteroides acidifaciens
Bacteroides caccae
Bacteroides uniformis
Parabacteroides
Parabacteroides distasonis
Mucispirillum schaedleri
p__Firmicutes f__[Mogibacteriaceae] s__Unc. [Mogibacteriaceae]
p__Firmicutes f__Lachnospiraceae s__Unc. Lachnospiraceae
p__Firmicutes f__Peptostreptococcaceae s__Unc. Peptostreptococcaceae
p__Firmicutes f__Ruminococcaceae s__Unc. Ruminococcaceae
[Ruminococcus] gnavus
Lactobacillus
Oscillospira
rc4−4
p__Firmicutes o__Clostridiales s__Unc. Clostridiales
p__Proteobacteria f__Enterobacteriaceae s__Unc. Enterobacteriaceae
Proteus
Sutterella
p__Proteobacteria o__RF32 s__Unc. RF32
Other taxa(25)
