# Supplementary figures and images for: Dietary Heme Induces Gut Dysbiosis, Aggravates Colitis, and Potentiates the Development of Adenomas in Mice
Source: Front Microbiol. 2017 Sep 21;8:1809. doi: 10.3389/fmicb.2017.01809 (PMC5613120; doi:10.3389/fmicb.2017.01809)

# CD vs. CD DSS

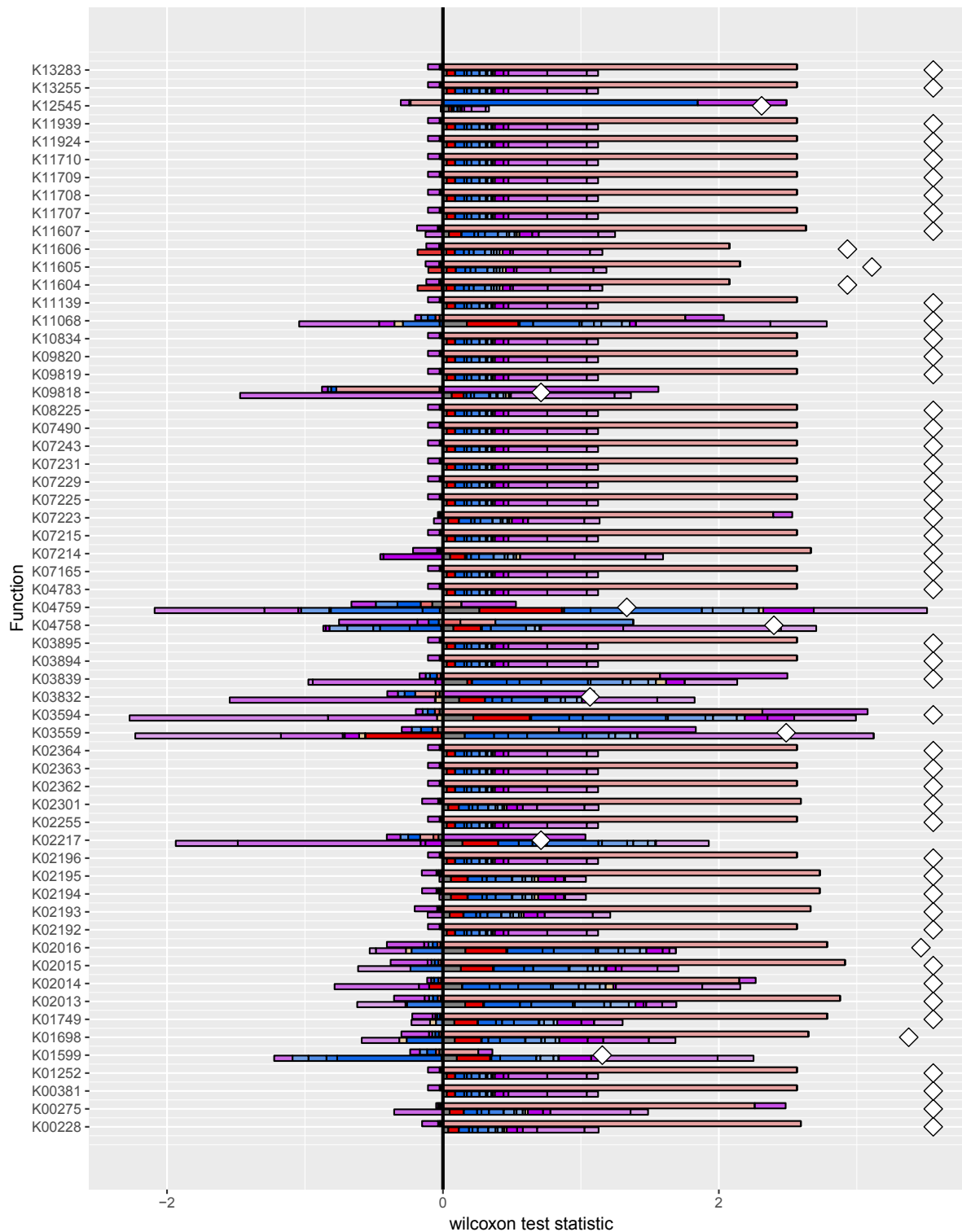

## Taxa

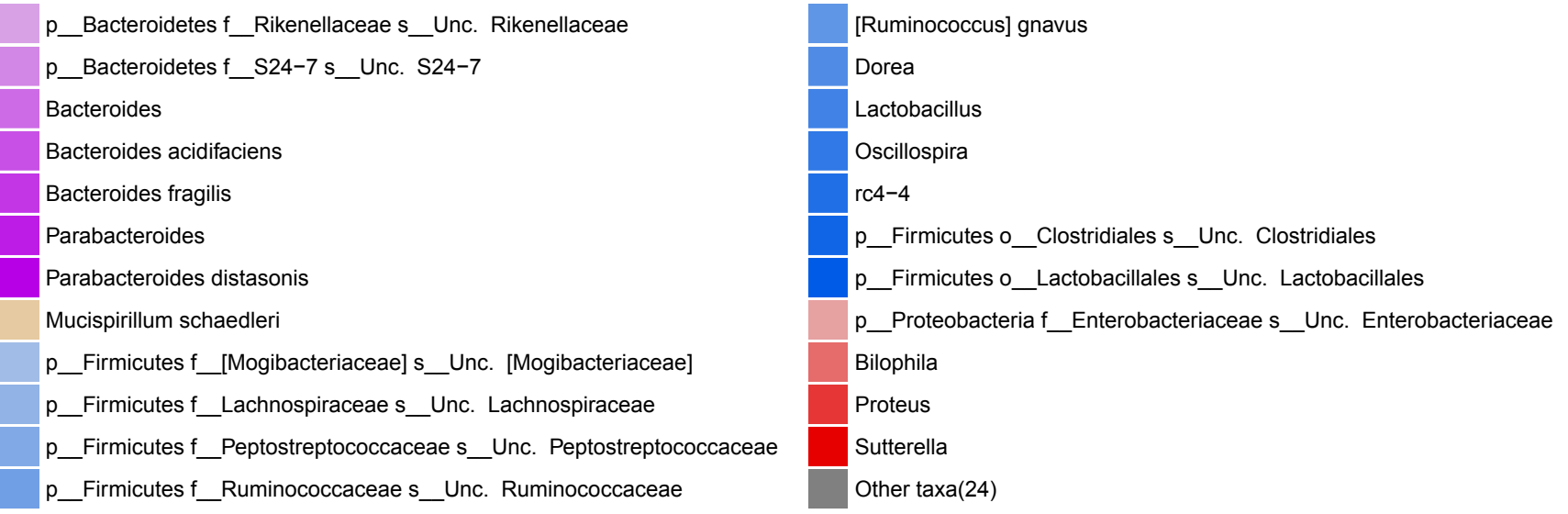

Supplement: FIGURE S2 — Taxon-level shift contribution profiles for several genes enriched by dextran sodium sulfate (DSS) determined by FishTaco. Mice were fed a control diet (CD) and received either water alone, or water with DSS (CD DSS) for 10 days (N = 8 mice per group). [file Image_2.PDF]
